# Association of Telehealth Reimbursement Parity With Contraceptive Visits During the COVID-19 Pandemic

**DOI:** 10.1001/jamanetworkopen.2022.6732

**Published:** 2022-04-11

**Authors:** Jacqueline Ellison, Megan B. Cole, Terri-Ann Thompson

**Affiliations:** 1 Department of Health Services, Policy, and Practice, Brown University School of Public Health, Providence, Rhode Island; 2 Department of Health Law, Policy, and Management, Boston University School of Public Health, Boston, Massachusetts; 3 Ibis Reproductive Health, Cambridge, Massachusetts

## Abstract

This cross-sectional study assesses the pattern of face-to-face and virtual contraceptive appointments in states in which insurers reimbursed both services equally vs states without a reimbursement parity policy.

## Introduction

The COVID-19 pandemic disrupted access to contraceptive care while increasing contraception need because fewer women planned to become pregnant.^1,2^ Increases in use of telehealth for contraception (telecontraception) during the pandemic present an opportunity to expand access to contraception via telehealth.^3^ Reimbursement parity mandates implemented by some states in response to COVID-19 required insurers to reimburse for telehealth at the same rate as for in-person services, potentially increasing access to telecontraception. These mandates may remove financial disincentives for offering remote services, but the implication of reimbursement parity for access to care during the pandemic has not been studied. We evaluated the association of state telehealth reimbursement parity mandates with remote and overall contraceptive encounters.

## Methods

This cross-sectional study used Symphony Health outpatient claims data to analyze a national sample of commercially insured female enrollees aged 14 to 49 years for the 10 months before (May 1, 2019-February 28, 2020) and during (March 1, 2020-December 31, 2020) the pandemic. Outcomes included total number of contraceptive visits (eTable 1 in the Supplement) and number of telecontraceptive visits (eTable 2 in the Supplement). Exposure was residency in 1 of 17 parity states (those that mandated commercial payer reimbursement parity for telehealth and in-person services).^4^ State policy data were obtained from the Kaiser Family Foundation and Centers for Disease Control and Prevention (eTable 3 in the Supplement).^4,5^ This study was approved by the Allendale Investigational Review Board, which waived the requirement for informed consent because only deidentified data were used, and followed the STROBE reporting guideline.

We used a difference-in-differences approach to estimate the adjusted change in contraceptive visits in parity vs nonparity states (those that did not mandate reimbursement parity) before and during the pandemic. We used negative binomial regression, adjusting for state and month fixed effects, patient age, contraceptive method, and whether the state issued a stay-at-home order or other executive actions to expand telehealth access. SEs were clustered at the state level. The offset was number of contraceptive visits per month-state for the telecontraceptive model and number of female enrollees of reproductive age in each state for the total contraceptive model. Analyses were conducted using Stata 15.1, with 2-tailed significance set at α = .05. Additional details are included in the eMethods in the Supplement.

## Results

The sample included 9 279 294 contraceptive claims among 34 109 287 enrollees. The Figure illustrates a steep decrease and increase in the proportion of enrollees with contraceptive and telecontraceptive visits, respectively, in the months after the pandemic began. There were no significant differences in prepandemic patterns.

**Figure.**
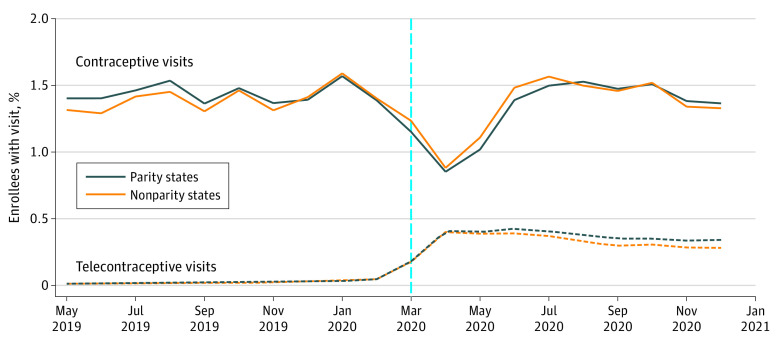
Unadjusted Monthly Patterns in the Percentage of Enrollees With a Contraceptive or Telecontraceptive Visit in States That Mandated vs Did Not Mandate Telehealth Reimbursement Parity Denominator for both total contraceptive and telecontraceptive visits is the number of female enrollees of reproductive age (14-49 years) in parity and nonparity states. Parity states included Arizona, Alaska, California, Delaware, Illinois, Iowa, Maine, Massachusetts, Montana, New Hampshire, New Jersey, New Mexico, New York, Rhode Island, Texas, Vermont, and Washington. All other states were nonparity states.

Telehealth accounted for 1.5% of contraceptive visits in all states before the pandemic and for 30.5% in parity states and 21.6% in nonparity states during the pandemic. In adjusted difference-in-differences models, telecontraceptive visits increased 25% in parity states (incidence rate ratio [IRR], 1.25; 95% CI, 1.01-1.55; *P* = .04) vs nonparity states (Table). There was no significant difference in total contraceptive visits between parity and nonparity states (IRR, 0.99; 95% CI, 0.93-1.07; *P* = .98).

**Table.** Total Contraceptive and Telecontraceptive Visits in States That Mandated vs Did Not Mandate Telehealth Reimbursement Parity Before vs During the COVID-19 Pandemic^a^

	Mean monthly visits		Absolute change, No. (%)	Adjusted difference-in-differences, IRR (95% CI)
	Before pandemic (May 2019-February 2020)	During pandemic (March-December 2020)		
**Telecontraceptive visits**				
Nonparity states	39.35	521.74	482.39 (1225.81)	1.25 (1.01-1.55)
Parity states	65.14	1153.50	1088.36 (1670.83)	
**Total contraceptive visits**				
Nonparity states	2549.83	2419.27	−130.56 (−5.12)	0.99 (0.93-1.07)
Parity states	4190.03	3776.35	−413.68 (−9.87)	

Abbreviation: IRR, incident rate ratio.

^a^ Adjusted models included age category (14-17, 18-25, 26-34, or 35-49 years); whether the method used was a long-acting reversible contraception (LARC) method; and 2 interaction terms with an indicator for before vs during the pandemic: (1) whether the state implemented a mandatory stay-at-home order, and (2) whether the state took any other executive action expanding telehealth. State and calendar month fixed effects were also included, and SEs were clustered at the state level.

## Discussion

Findings demonstrated an increase in telecontraceptive visits in states that mandated reimbursement parity vs states that did not. This increase did not correspond to an increase in total contraceptive encounters, suggesting that, although reimbursement parity may have expanded access to telehealth options, it did not improve access to contraception overall.

One limitation is that we could not capture changes in the number of enrollees with commercial coverage over the study period, which, as with other COVID-related shifts in contraceptive supply or demand, could bias results if differentially associated with service use in parity and nonparity states. Insurers in nonparity states may have implemented parity anyway, in which case our estimates are conservative. Ultimately, the findings highlighted how telehealth reimbursement parity policies alone were insufficient in addressing unmet need for contraceptive care.

## Notes

Supplement. **eTable 1.** Diagnostic Codes Used to Identify Contraceptive Encounters **eTable 2.** Billing Codes Used to Identify Telehealth Encounters **eTable 3.** State Policy Actions Implemented in Response to COVID-19
eMethods.
