# Supplementary material for: Association of Telehealth Reimbursement Parity With Contraceptive Visits During the COVID-19 Pandemic
Source: JAMA Netw Open. 2022 Apr 11;5(4):e226732. doi: 10.1001/jamanetworkopen.2022.6732 (PMC9002337; doi:10.1001/jamanetworkopen.2022.6732)
Supplement: Supplement. — eTable 1. Diagnostic Codes Used to Identify Contraceptive Encounters eTable 2. Billing Codes Used to Identify Telehealth Encounters eTable 3. State Policy Actions Implemented in Response to COVID-19 eMethods. [file jamanetwopen-e226732-s001.pdf]

Supplementary Online Content

Ellison J, Cole MB, Thompson TA. Association of telehealth reimbursement parity with contraceptive visits during the COVID-19 pandemic. *JAMA Netw Open*. 2022;5(4):e226732. doi:10.1001/jamanetworkopen.2022.6732

eTable 1. Diagnostic Codes Used to Identify Contraceptive Encounters

eTable 2. Billing Codes Used to Identify Telehealth Encounters

eTable 3. State Policy Actions Implemented in Response to COVID-19

eMethods.

This supplementary material has been provided by the authors to give readers additional information about their work.

© 2022 Ellison J et al. *JAMA Network Open.*

eTable 1. Diagnostic Codes Used to Identify Contraceptive Encounters

ICD-10 Code	Definition	LARC[1]
Z30	Encounter for contraceptive management	No
Z300	Encounter for general counseling and advice on contraception	No
Z3001	Encounter for initial prescription of contraceptives	No
Z30011	Encounter for initial prescription of contraceptive pills	No
Z30012	Encounter for prescription of emergency contraception	No
Z30015	Encounter for initial prescription of vaginal ring hormonal contraceptive	No
Z30016	Encounter for initial prescription of transdermal patch hormonal contraceptive device	No
Z30014	Encounter for initial prescription of intrauterine contraceptive device	Yes
Z30013	Encounter for initial prescription of injectable contraceptive	Yes
Z30017	Encounter for initial prescription of implantable subdermal contraceptive	Yes
Z30018	Encounter for initial prescription of other contraceptives	No
Z30019	Encounter for initial prescription of other contraceptives unspecified	No
Z3002	Counseling and instruction in natural family planning to avoid pregnancy	No
Z3009	Encounter for other general counseling and advice on contraception	No
Z304	Encounter for surveillance of contraceptives	No
Z3040	Encounter for surveillance of contraceptives unspecified	No
Z3041	Encounter for surveillance of contraceptive pills	No
Z3044	Encounter for surveillance of vaginal ring hormonal contraceptive device	No
Z3045	Encounter for surveillance of transdermal patch hormonal contraceptive device	Yes
Z3043	Encounter for surveillance of intrauterine contraceptive device	Yes
Z3042	Encounter for surveillance of injectable contraceptive	Yes
Z3046	Encounter for surveillance of implantable subdermal contraceptive	Yes
Z30014	Encounter for initial prescription of intrauterine contraceptive device	Yes
Z30430	Encounter for insertion of intrauterine contraceptive device	Yes
Z30431	Encounter for routine checking of intrauterine contraceptive device	Yes
Z30432	Encounter for removal of intrauterine contraceptive device	Yes
Z3049	Encounter for surveillance of other contraceptives	No
Z308	Encounter for other contraceptive management	No
Z309	Encounter for contraceptive management, unspecified	No
Z30433	Encounter for removal and reinsertion of intrauterine contraceptive device	Yes

LARC, long-acting reversable contraceptive

[1]*Adjusted models distinguish between contraceptive encounters for long-acting and short-acting methods because long-acting contraceptives typically require an in-person visit. We adjust for, rather than exclude encounters for long-acting methods because some could also be delivered remotely (e.g., encounter for surveillance of implantable subdermal contraceptive) and because we are interested in the effect of reimbursement parity on overall contraceptive encounters in addition to telehealth visits.*

© 2022 Ellison J et al. *JAMA Network Open.*

**eTable 2.** Billing Codes Used to Identify Telehealth Encounters[1]	
CPT/HCPCS codes	Definition
99441, 99442, 99443	Telephone evaluation and management
99421, 99422, 99423, 99444, 98970, 98971, 98972	Online digital evaluation and management
98966-98969, G2010, G2012, G9868-G9870, S9110, G0071	Other

CPT, current procedural technology; HCPCS, healthcare common procedure coding system

[1]In addition to CPT and HCPCS codes, the 'place of service' code corresponding to telehealth (specific to the Symphony health database). These data did not include procedure code modifiers. Consequently, telehealth visits that were billed using traditional evaluation and management codes are not captured if they did not also have a place of service code indicating remote service delivery.

© 2022 Ellison J et al. *JAMA Network Open.*

**eTable 3.** State Policy Actions Implemented in Response to COVID-19	
Mandated reimbursement parity for telehealth and in-person services[1]	AZ, AK, CA, DE, IL, IA, ME, MA, MT, NH, NJ, NM, NY, RI, TX, VT, WA
Required commercial insurers to expand access to telehealth[2]	AK, AZ, AK, CA, CO, CT, DE, HI, ID, IL, IN, IA, KS, KY, LA, ME, MD, MA, MI, MS, MT, NE, NH, NJ, NM, NY, NC, ND, OH, OR, OK, RI, SD, TX, UT, VT, VA, WA
Mandatory stay-at-home order[3]	AL, AK, AZ, AR, CA, CO, CT, DE, FL, GA, HI, ID, IL, IN, KS, LA, ME, MD, MI, MN, MS, MO, MT, NE, NV, NH, NJ, NC, ND, OH, OR, PA, RI, SC, TN, VT, VA, WA, WV, WI, WY

[1] *Enacted laws requiring provider reimbursement parity between telehealth and in-person services. Some states may have had reimbursement parity prior to the COVID-19 outbreak, but for the purposes of our study were only included if there was an executive action released in response to the pandemic to remind insurers of these requirements*

[2] *Took any executive action mandating state-regulated commercial plans to expand access to telehealth (e.g., coverage parity, reimbursement parity, waiving or limiting cost-sharing, or expanding the telehealth modalities covered)*

[3] *Implemented a mandatory stay-at-home order for all state residents from March – April 2020*

© 2022 Ellison J et al. *JAMA Network Open.*

eMethods.

Both telecontraceptive visits and total contraceptive visits were measured monthly, and analyses were conducted using aggregated data at the level of state, month, age category, and method, where method represents whether the encounter was for a long-acting reversible contraception (LARC; intrauterine device, subdermal implant, or shot) or a method or service more commonly delivered remotely (pill, patch, ring, or counseling).

Among 34 109 287 reproductive-aged female enrollees, 4 424 293 had a contraceptive encounter before the pandemic (5/2019-2/2020) and 4 188 396 had a contraceptive encounter during the pandemic (3/2020-12/2020). Of these, 69 693 visits were delivered remotely before the pandemic, and 1 045 181 were delivered during the pandemic. Over the study period, enrollees aged 18 to 25 years were most likely to have a contraceptive encounter (1.2%), and among enrollees with any contraceptive encounter, those aged 14 to 17 years were most likely to have a telecontraceptive visit (13.8%). There were no major differences in the age of enrollees using contraception or telecontraception between states with vs without reimbursement parity.

© 2022 Ellison J et al. *JAMA Network Open.*
